# Two Worlds

**DOI:** 10.3201/eid0501.AC0501

**Published:** 1999

—Michael Eather and Friends

This issue was originally published without an accompanying cover story. 

**Figure:**
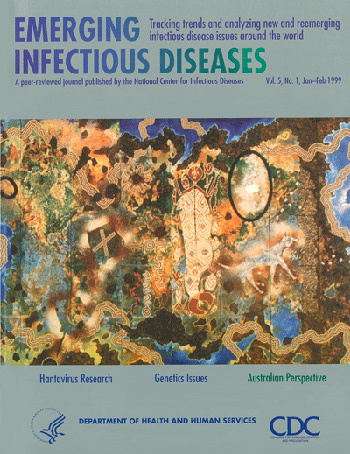
Michael Eather (b. 1963) and Friends, Queensland, Australia. Two Worlds. Synthetic polymer paint, natural pigments, shellac, and photocopied images on 75 boards. Celebrating the Queensland Art Gallery Centenary 1895-1995.

